# Supplementary material for: Blood parasites in Passeriformes in central Germany: prevalence and lineage diversity of Haemosporida (Haemoproteus, Plasmodium and Leucocytozoon) in six common songbirds
Source: PeerJ. 2019 Jan 31;6:e6259. doi: 10.7717/peerj.6259 (PMC6360073; doi:10.7717/peerj.6259)
Supplement: Table S1 [file peerj-07-6259-s001.docx]

**Blood parasites in Passeriformes in central Germany: Prevalence and lineage diversity of Haemosporida (*Haemoproteus*, *Plasmodium* and *Leucocytozoon*) in six common songbirds**

Yvonne R. Schumm, Christine Wecker, Carina Marek, Mareike Wassmuth, Anna Bentele, Hermann Willems, Gerald Reiner and Petra Quillfeldt

**Supplemental Material**

Table S1: BLAST results of samples that could not be assigned to a single Haemosporida lineage.

| **Parasite** | **Lineage name (MalAvi)** | **Samples (n)** | **Match (%)** | **Sample ID (species)** |
| --- | --- | --- | --- | --- |
| *Haemoproteus* spp. | PARUS10, PARUS1 | 1 | 95 | NK18_A40 (*P. major*) |
| *Leucocytozoon* spp*.* | PARUS74; PARUS19 | 1 | 100 | NK15_139 (*P. major*) |
|  | PARUS76; PARUS74; PARUS72 | 1 | 93 | NK15_16 (*P. major*) |
|  | PARUS81; PARUS70 | 1 | 93 | NK15_161 (*P. major*) |
|  | PARUS82; PARUS77; PARUS4; PARUS26; PARUS18; PERATE06 | 1 | 97 | NK15_18 (*P. major*) |
|  | PARUS82; PARUS4 | 1 | 98 | NK15_27 (*P. major*) |
|  | PARUS74; PARUS72 | 1 | 97 | NK15_23 (P. major) |
|  | PARUS77; PARUS4; PARUS26 | 1 | 99 | NK15_83 (P. major) |
|  | PYCGPI03; PARUS82; PARUS77; PARUS73; PARUS4; PARUS18; ERYVIR01; COCOR19 | 1 | 95 | NK18_A06 (P. major) |
|  | SYBOR22; PARUS74; PARUS72; CYACAE03; AFR215 | 1 | 99 | NK18_A20 (P. major) |
|  | SYCON05; CYACAE03 | 1 | 98 | NK18_A39 (*P. major*) |
